# Equine “Idiopathic” and Infundibular Caries-Related Cheek Teeth Fractures: A Long-Term Study of 486 Fractured Teeth in 300 Horses

**DOI:** 10.3389/fvets.2021.646870

**Published:** 2021-05-28

**Authors:** Padraic Martin Dixon, Rebekah Kennedy, Richard J. M. Reardon

**Affiliations:** The Royal (Dick) School of Veterinary Studies and The Roslin Institute, The University of Edinburgh, Edinburgh, United Kingdom

**Keywords:** horse, equine dental disease, equine dental fractures, equine idiopathic cheek teeth fractures, infundibular caries-related dental fracture

## Abstract

**Background:** Limited objective information is available on the prevalence of non-traumatic equine cheek teeth fractures, the signalment of affected horses, and the clinical features and treatment of these fractures.

**Objectives:** This study aims to document patterns of idiopathic and infundibular caries-related cheek teeth fractures in a referral population and evaluate associations between fracture patterns and horse age, Triadan position of affected teeth, clinical signs, and deemed necessity for treatment.

**Study Design:** A retrospective case review.

**Methods:** The clinical records at Edinburgh University Veterinary School (2010–2018) were examined for the presence of non-traumatic equine cheek teeth fractures. Variations in the frequencies of different fracture patterns were compared between horse ages, Triadan tooth positions, clinical signs, and deemed necessity for treatment.

**Results:** Records of 300 horses with 486 non-traumatic cheek teeth fractures including 77% maxillary and 23% mandibular teeth with a mean of 1.6 (range 1–10) fractured teeth/horse were available. Fracture patterns included maxillary first and second pulp horn (“slab”) cheek teeth fractures (*n* = 171), caries-related infundibular fractures (*n* = 88), other maxillary teeth fracture patterns (*n* = 92), mandibular first and second pulp horn (“slab”) fractures (*n* = 44), other mandibular fracture patterns (*n* = 62), and complete clinical crown loss (*n* = 29; including 23 maxillary and 6 mandibular teeth). The median age of affected horses ranged from 11 years with maxillary “slab” fractures to 15 years with infundibular caries-related fractures. Triadan 08–10s were the most commonly (86%) fractured maxillary teeth. The Triadan 08 and 09 positions were the most commonly (64%) fractured mandibular teeth. No clinical signs were noted in horses with 48% of the fractured teeth; oral pain/quidding was recorded with 26%, clinical apical infection with 23%, and bitting/headshaking problems with 6%. Treatments included extraction of 40% fractured teeth, extraction of small/loose fragments (10%), and odontoplasty. Stable remnants of 60% of fractured teeth were left in horses without clinical signs.

**Main Limitations:** Long-term follow-up information was not available for all cases.

**Conclusions:** There is increasing recognition of equine non-traumatic cheek teeth fractures, with about half not causing clinical signs. Teeth with apical infection, multiple fractures, or advanced caries require extraction. Other fractured teeth with subclinical endodontic disease may not need exodontia unless they later cause clinical signs.

## Introduction

Most equine cheek teeth fractures occur in the absence of known evidence of trauma and have been termed *idiopathic* cheek teeth fractures (1). These fractures most commonly affect the maxillary cheek teeth, especially the Triadan 09 position (1–4), and a pathological study showed 25% of these teeth to have had chronic endodontic disease prior to fracturing (2). More recently, a subset of these fractures, i.e., midline sagittal fractures of maxillary cheek teeth, has been recognized to be caused by coalescence of deeply carious infundibula (5–7), and these have now been termed *infundibular caries-related (sagittal) fractures* (8).

The fracture planes in the remaining non-traumatic (idiopathic) cheek teeth fractures always run through one or more pulp horns causing direct pulpar exposure (i.e., are *complicated* dental fractures) in a variety of patterns (2). The most common pattern, colloquially termed a *slab* or *buccal slab* fracture, is a thin sagittal buccal fracture of the clinical crown only, through the first and second (buccal) pulp horns of maxillary teeth (1–4, 9). A variety of other, less common maxillary teeth fracture planes, involving one or more pulp horns, can also occur (1–4).

Idiopathic fractures less commonly occur in mandibular cheek teeth, where the most prevalent pattern is also a sagittal (“slab”) fracture through the first and second pulp horns (1–4). Because mandibular cheek teeth do not have infundibula, this fracture plane lies closer to the sagittal midline and these fractures can clinically appear as “midline” sagittal fractures. A variety of other fracture planes through pulp horns occur less commonly (1–4).

Fracture sites can fill with food, which, along with masticatory movements, displaces smaller or more mobile dental fragments which can cause lingual or buccal lacerations, in addition to overstretching the periodontal ligaments. These soft tissue insults can cause oral pain with resultant quidding, loss of appetite, and bitting and headshaking problems. Spontaneous loss or extraction of displaced or mobile fragments usually resolves the oral pain (3, 4).

If a fracture-related pulpar exposure is not sealed off with tertiary dentine, pulpar, or apical infection may develop causing clinical signs such as unilateral nasal discharge and maxillary or mandibular swellings. Some fractured cheek teeth not causing clinical signs of apical infection have evidence of apical infection and alveolar remodeling on radiography (authors' unpublished observations). Rowley et al. (10) showed that 77% of all such fractured cheek teeth had apical changes on computed tomographic (CT) imaging. Due to continued dental eruption and wear, and in the absence of any further subocclusal secondary dentine deposition, occlusal pulpar exposure may later become apparent in some remaining non-fractured pulps, confirming death of these exposed pulp(s). In contrast, the exposed pulp horns in other fractured teeth become fully sealed off by tertiary dentine, and apparently, normal dental function continues (authors' unpublished observations).

## Materials and Methods

The clinical and imaging records of horses undergoing dental examination (some on multiple occasions over many years) at The University of Edinburgh Equine Hospital between 2010 and 2018 were retrospectively examined for the presence of non-traumatic cheek teeth fractures, i.e., where there was no history or clinical evidence of these fractures being due to external trauma or iatrogenic damage. Horses with fissure (hairline) cheek teeth fractures were not included in this study. Horses that had other major dental-related disorders (such as a dental sinusitis or oro-maxillary fistulas unrelated to a fractured tooth, or severe diastemata-related periodontal disease) were excluded, to allow meaningful assignment of clinical signs to any detected fractured teeth. In horses with more than one fractured cheek tooth, the more generalized clinical signs such as quidding or headshaking were assigned to every fractured tooth present at that examination if there was no clear evidence to assign the clinical signs to one tooth, such as an acute onset oral pain/quidding caused by a recent maxillary cheek tooth “slab fracture.” More specific clinical signs such as unilateral nasal discharge due to dental sinusitis or swellings of the supporting bones were only attributed to the teeth shown to have clinical apical infection by detailed clinical and imaging examinations.

The recorded treatment for fractured cheek teeth that were examined more than once was the last treatment given for that dental fracture. For example, a fractured tooth may have initially been treated by extraction of displaced and/or mobile fragments present (and usually odontoplasty of sharp edges of the fractured and possibly of the adjacent and opposing teeth). However, if clinical signs of apical infection or further fractures developed later, the teeth were then fully extracted, and the latter was the recorded treatment for the purpose of this study.

### Statistical Analyses

Data were summarized and presented as mean/median and ranges. Normality of variables was assessed (where appropriate) graphically using Shapiro–Wilks tests. Non-parametric tests were used for variables that were not normally distributed.

The association between fracture type and age was assessed using a Kruskal–Wallis rank sum test, followed by pairwise comparisons between fracture types using Wilcoxon rank sum tests.

Chi-squared tests were used to evaluate the associations between Triadan tooth position and frequency of infundibular caries-related fracture (maxillary teeth) or idiopathic fracture and between fracture type and frequency of tooth extraction.

A Fisher's exact test was used to evaluate the association between mandibular tooth position (explanatory variable) and frequency of any types of dental fractures (outcome variable). Odds ratios were calculated between groups using an unconditional maximum likelihood estimation, with small sample adjustment when group sizes were <5.

Univariable logistic regression models, with horse as a random effect, were produced to evaluate associations between the outcomes “oral pain” (that for the purpose of this analysis included all horses with oral pain/quidding and bitting problems) and nasal discharge and the explanatory variable fracture types, while accounting for the potential effect of multiple fracture types in the same horse.

Statistical analyses were performed in RStudio™. Significance was set as *P* < 0.05.

## Results

### Numbers of Fractured Teeth per Case

Complete clinical records of 300 horses with 486 non-traumatic cheek teeth fractures [374 (77%) maxillary and 112 (23%) mandibular teeth] were available with a mean of 1.6 (range 1–10) fractured teeth per horse. A single fractured tooth was present in 206 horses: two in 59 horses, three in 16 horses, four in 10 horses, five in 5 horses, six in 3 horses, seven in 3 horses, and 10 fractured teeth in 1 horse. Long-term follow-up information was collected by a written questionnaire (in a separate exodontia study) only on cases that had the fractured teeth extracted a mean of 5.9 years (standard deviation 3.1 years) following extraction. Informal follow-up information was collected on some other cases.

### Fracture Patterns

#### Infundibular Caries-Related Cheek Tooth Fractures

**Figure 1:**
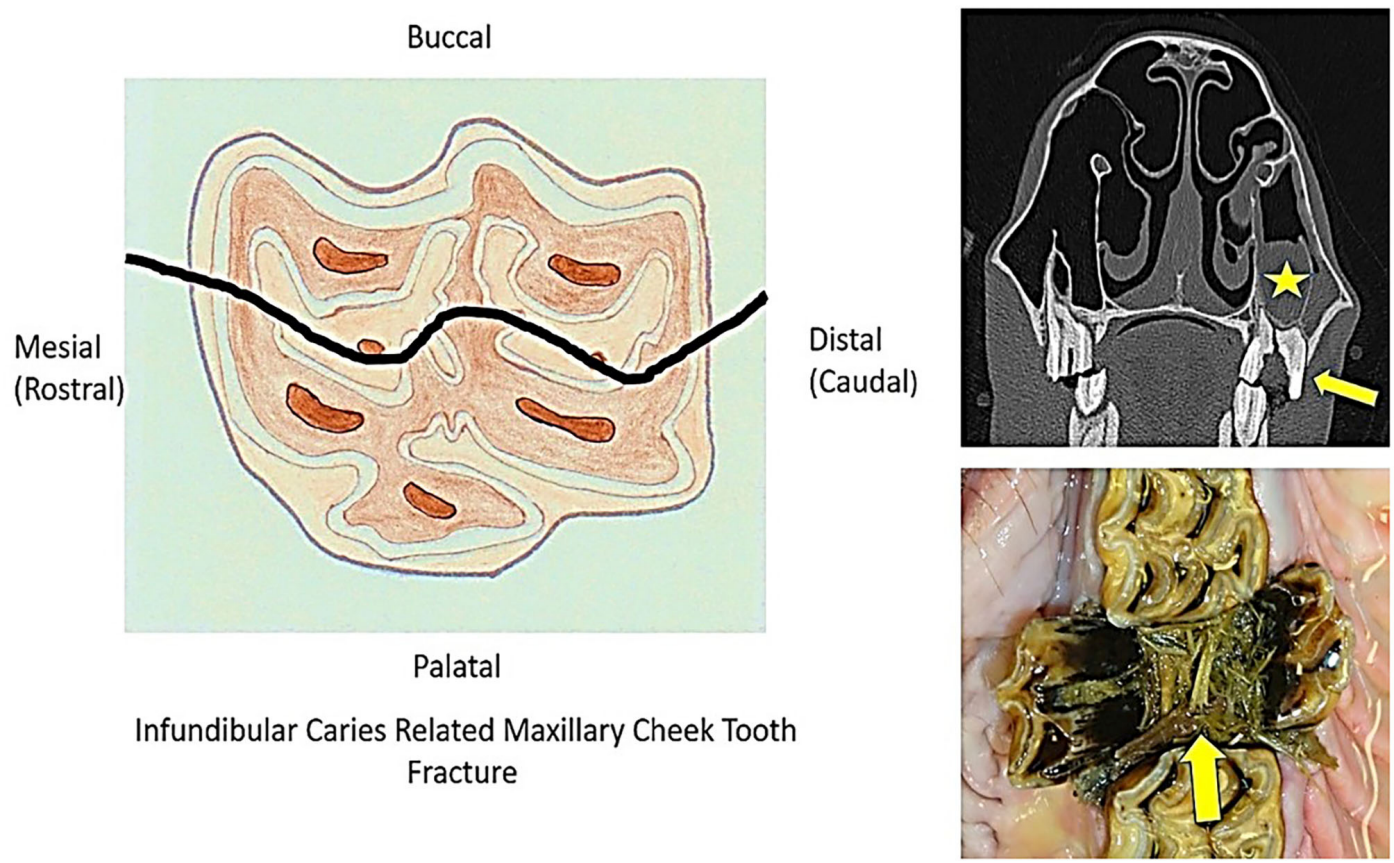
The diagram on the left shows the fracture plane through both infundibula (that would have advanced caries and likely coalescence of both infundibula before fracturing). The top right image shows a transverse computed tomographic image of an aged Triadan 209 with a midline infundibular caries-related sagittal fracture (arrow) with resultant apical infection and maxillary sinusitis (star). The bottom right image shows a postmortem image of an infundibular caries-related midline sagittal Triadan 109 fracture with palatal and buccal displacement of the two fracture fragments and food impaction in the fracture site (arrow).

Infundibular caries-related (midline) sagittal fractures were present in 88 (18.1% of fractured teeth) maxillary cheek teeth, with both fragments present in 84/88 (95%) teeth and a single fragment present in 4/88 (5%) teeth (Figure 1).

#### Maxillary First and Second Pulp Horn (“Slab”) Fractures

**Figure 2:**
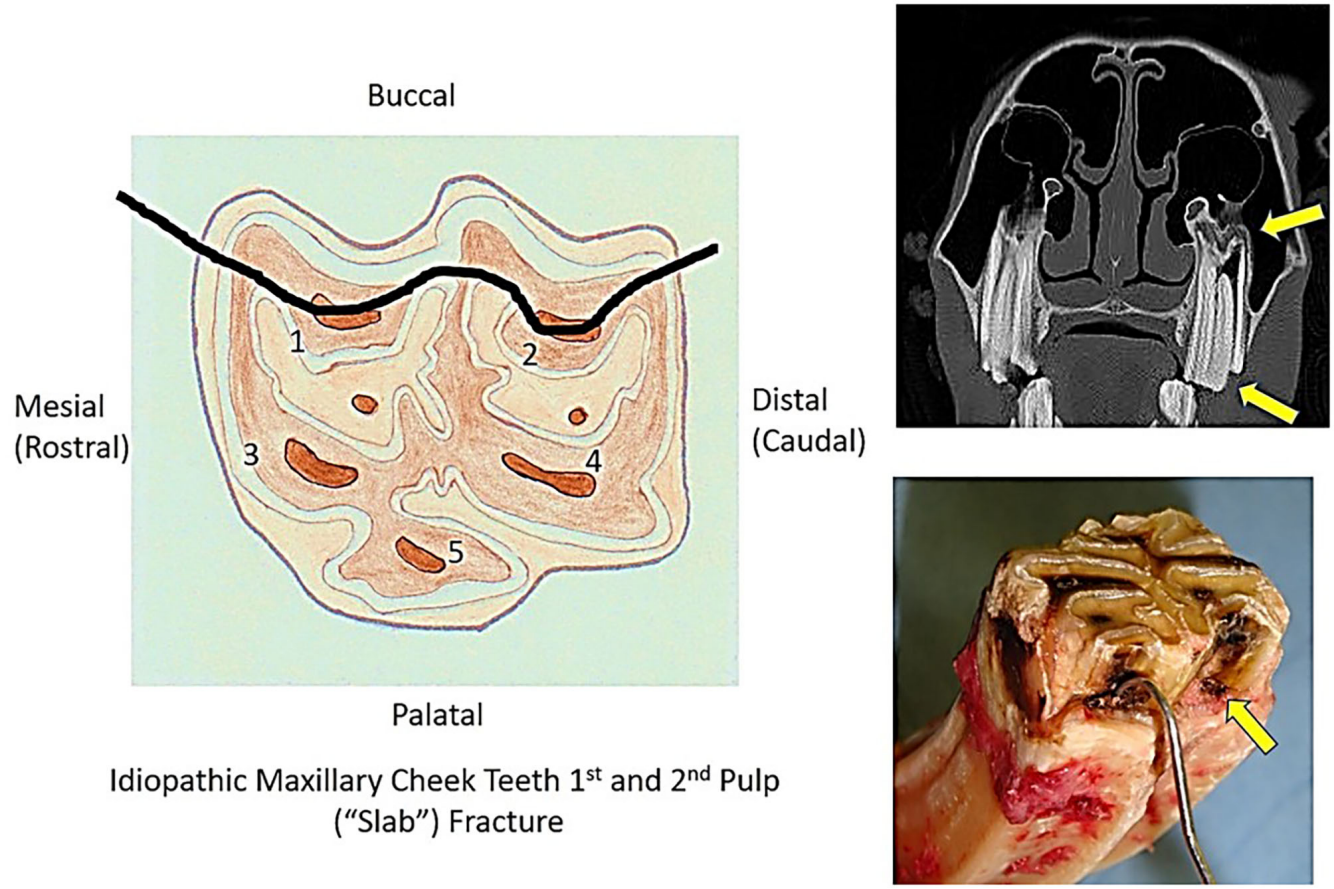
The diagram on the left shows the maxillary cheek teeth pulp horn positions and the site of a fracture plane through pulp horns 1 and 2 (buccal “slab” fracture). Top right is a transverse computed tomographic image of a young Triadan 209 with a buccal “slab” fracture (bottom arrow). There is a resultant apical infection as evidenced by hypoattenuation of an affected pulp horn and, more so, of the common pulp chamber. The affected alveolus and the overlying maxillary sinus mucosa are remodeled and swollen, respectively (top arrow). The bottom left image shows a fractured maxillary cheek tooth extracted because of clinical apical infection. It has occlusal pulpar exposure of the first pulp horn (containing probe) and the second pulp horn (arrow).

The most common maxillary (and overall) cheek teeth fracture pattern was a fracture of the clinical crown only, through the first and second pulp horns (“slab” or “buccal slab” fracture) (*n* = 171; 35.2% of all fractures) (Figure 2). The smaller buccal fragments were missing in 162/171 (95%) cases, and horses that had recently lost the buccal fragment had adjacent gingival damage and usually buccal ulceration.

#### Miscellaneous Other (“Atypical”) Maxillary Cheek Teeth Fractures

**Figure 3:**
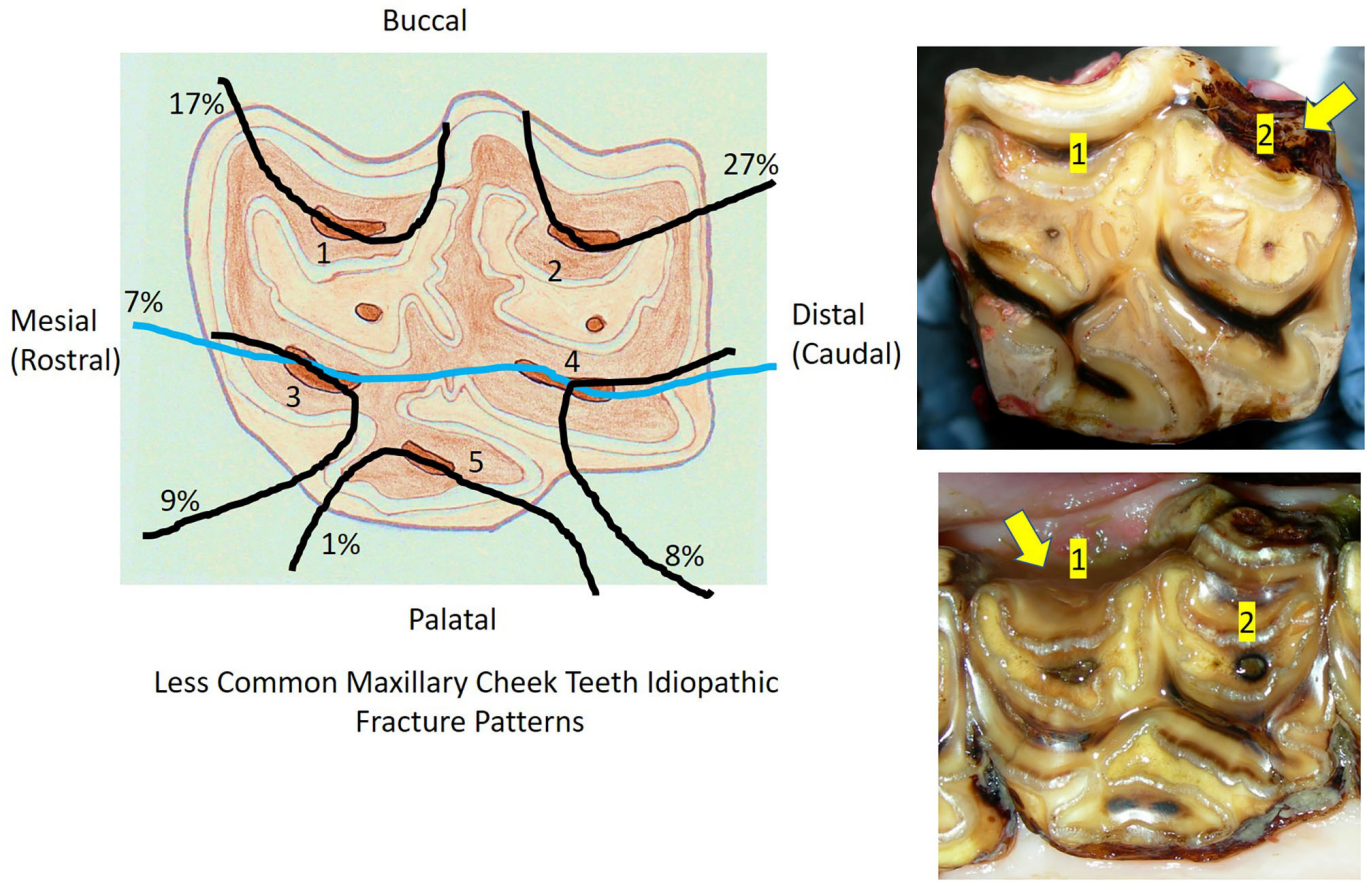
The diagram on the left shows the six most common idiopathic maxillary cheek teeth fracture planes other than “slab” fractures (i.e., “atypical fractures”) and the proportions of each fracture plane in this category, with five patterns involving single pulp horns. The top right image shows a tooth with a second pulp horn fracture and the bottom right image shows a tooth with a first pulp horn fracture.

Twenty-eight other different (“atypical”) patterns of maxillary cheek teeth fractures were recognized in 92 teeth that had some remaining clinical crown, usually with loss of smaller fracture fragments. The six most commonly recognized atypical fracture patterns are shown in Figure 3. These fractures usually involved single pulp horns, including pulp horn 1 (*n* = 16; 17% of identified atypical fracture patterns), pulp horn 2 (*n* = 25; 27%), pulp horn 3 (*n* = 8; 9%), pulp horn 4 (*n* = 7; 8%), pulp horn 5 (*n* = 1; 1%), and pulp horn 6 (*n* = 1; 1%). Two pulp horns were fractured in 13 teeth with 7 different fracture patterns, 3 pulps in 6 teeth with 5 different patterns, and 4 pulp horn fractures in 2 teeth with 2 different patterns. Eight teeth had different combinations of fractures through pulp horns and a single infundibulum. Five other teeth were classified as “atypical maxillary” in the records without further identification of their fracture pattern. Overall, the buccal pulps were most commonly involved, with various combinations of fractures of the first pulp horn present in 39 teeth, of the second pulp horns in 46 teeth, of the third pulp horn fractures in 30 teeth, of the fourth pulp horns in 32 teeth, and of the fifth pulp horns in 23 teeth.

#### Mandibular First and Second Pulp Horn (“Slab”) Fractures

**Figure 4:**
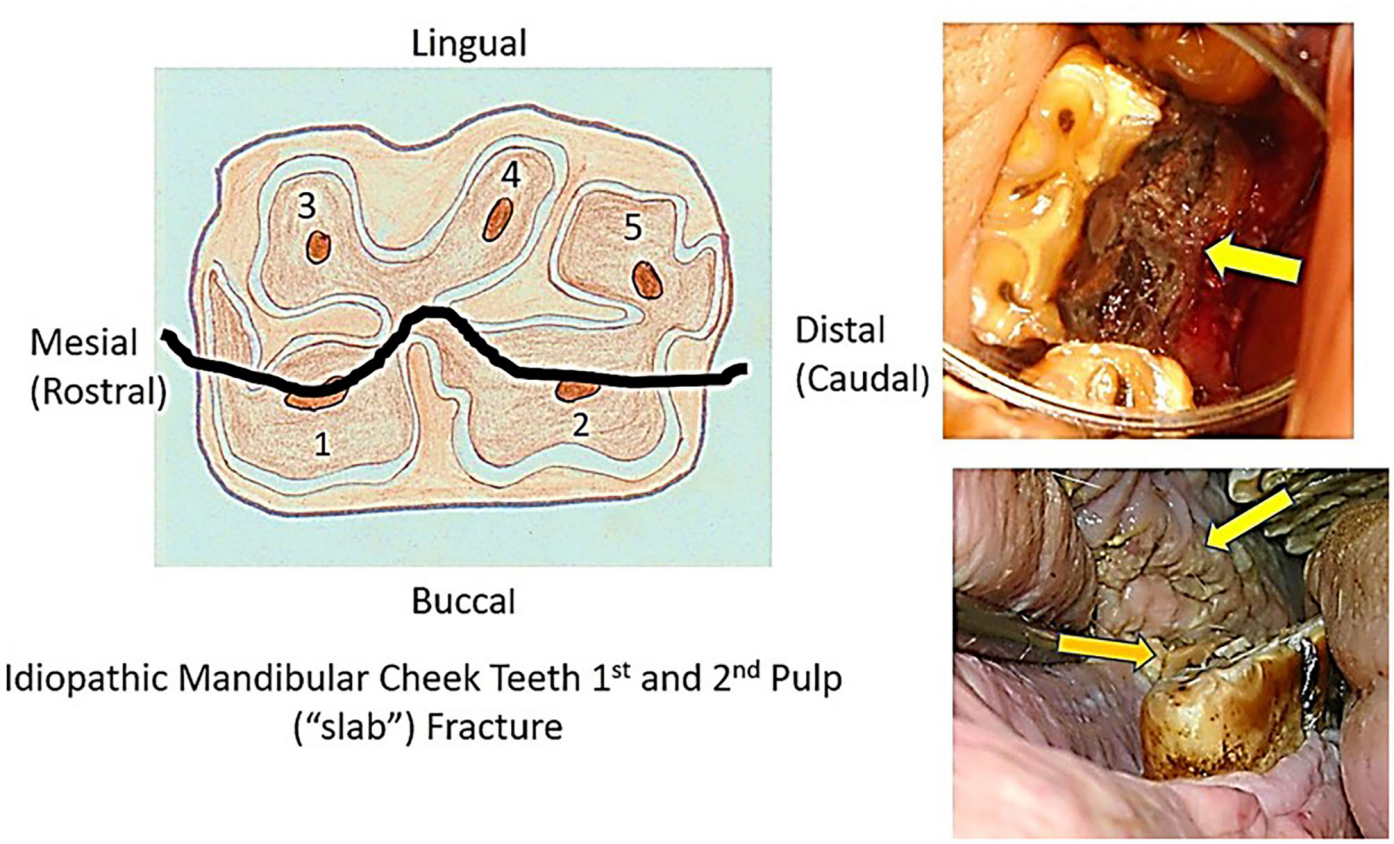
The diagram on the left shows the mandibular cheek teeth pulp horn positions and the site of a fracture plane through pulp horns 1 and 2 (“slab” fracture). The top right image shows a left mandibular cheek tooth with a first and second pulp horn fracture and loss of the buccal fragment (arrow) and slight lingual displacement of the remaining tooth. The bottom right image shows a displaced buccal fragment of a “slab” fracture (bottom arrow) that has caused marked, chronic buccal trauma (top arrow).

The most common fracture pattern in mandibular cheek teeth was also a fracture through the first and second pulp horn (“slab”) fractures (*n* = 44), often extending into the reserve crown and with some appearing more like a midline sagittal fracture (Figure 4).

#### Miscellaneous Other (“Atypical”) Mandibular Cheek Teeth Fractures

**Figure 5:**
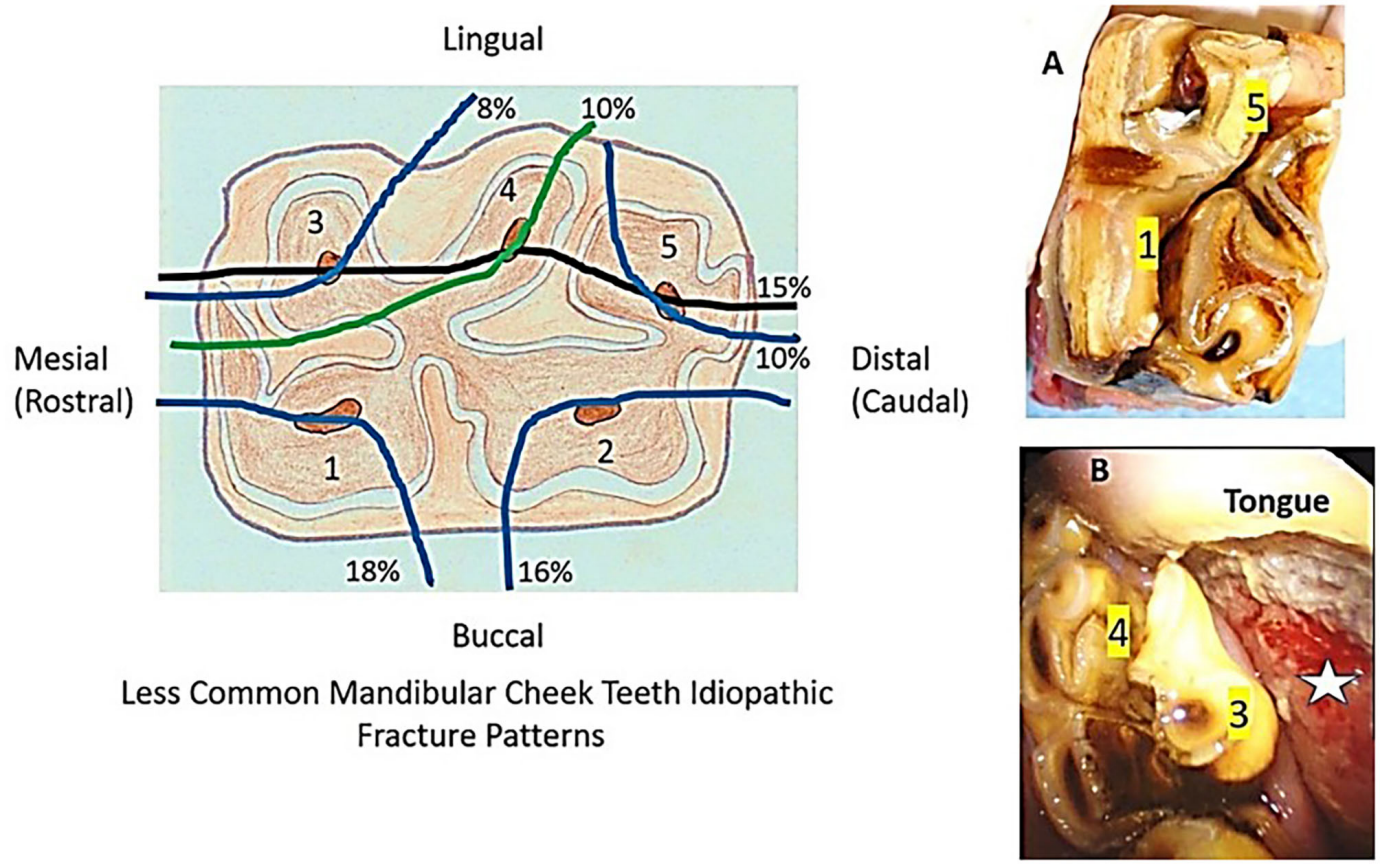
The diagram on the left shows the six most common idiopathic mandibular cheek teeth fracture planes (five through single pulp horns) other than slab fractures (i.e., “atypical fractures”) and the proportions of each fracture plane in this category. The top right image **(A)** shows an extracted tooth with a fracture extending through the first and fifth pulp horns. The bottom right image **(B)** is an intra-oral view of an oblique fracture through the fourth pulp horn (similar to that shown in the diagram on the left). The smaller displaced fragment (containing the third pulp horn) remains attached by its periodontal and gingival attachments and has caused severe tongue ulceration (star).

Sixteen other fracture patterns were identified in 62 mandibular cheek teeth. A single pulp horn fracture was present in 38 teeth involving pulp horn 1 (*n* = 11; 18% of the 62 fractures), pulp horn 2 (*n* = 10; 16%), pulp horn 3 (*n* = 5; 8%), pulp horn 4 (*n* = 6; 10%), and pulp horn 5 (*n* = 6; 10%). Two pulp horns were fractured in 10 teeth in seven different patterns, three pulp horns in 12 teeth in three different patterns (most commonly of pulps 3, 4, and 5: *n* = 9; 15%), and four pulp horns were fractured in 2 teeth in the same pattern (Figure 5).

#### Complete Loss of Maxillary or Mandibular Clinical Crowns

Fractures resulting in complete loss of clinical crown were present in 29 teeth, including 23 maxillary and 6 mandibular teeth. It was not possible to determine the previous fracture patterns prior to complete clinical crown loss in these teeth.

#### Age of Affected Horses

The mean, median, and range of ages of horses with different fracture patterns are presented in Table 1 with accurate data available for 286/300 horses. The median age of affected horses were in general similar (i.e., early in their second decade) and ranged from 11 years (maxillary “slab” pattern) to 15 years (infundibular caries-related fractures). Horses with infundibular caries-related fractures were significantly older (*P* < 0.001) than those with maxillary slab fractures. No other significant differences in ages were observed between groups.

**Table 1:** The mean, median, and range of ages (in years) for horses with six different cheek teeth fracture patterns.

**Age (years)**	**Infun caries**	**Max slab**	**Atypic max**	**Man slab**	**Atypic man**	**No clin crown**
No of teeth	88	171	92	44	62	29
Mean (sd)	14.1 (3.83)	11.9 (4.21)	13.5 (4.86)	13.2 (4.53)	13.2 (5.32)	12.8 (4.45)
Median	15	11	13	13	13	12.5
Range	4–26	2–25	4–27	6–22	3–28	6–20

*Infun caries, infundibular caries-related fracture; Max slab, maxillary first and second pulp horn fractures; Atypic max, atypical maxillary cheek teeth fracture patterns; Man slab, mandibular first and second pulp horn fractures; Atypic man, atypical mandibular cheek teeth fracture patterns; No Clin Crown, fracture with the absence of clinical crown*.

### Triadan Position of Fractured Teeth

#### Maxillary Teeth

**Figure 6:**
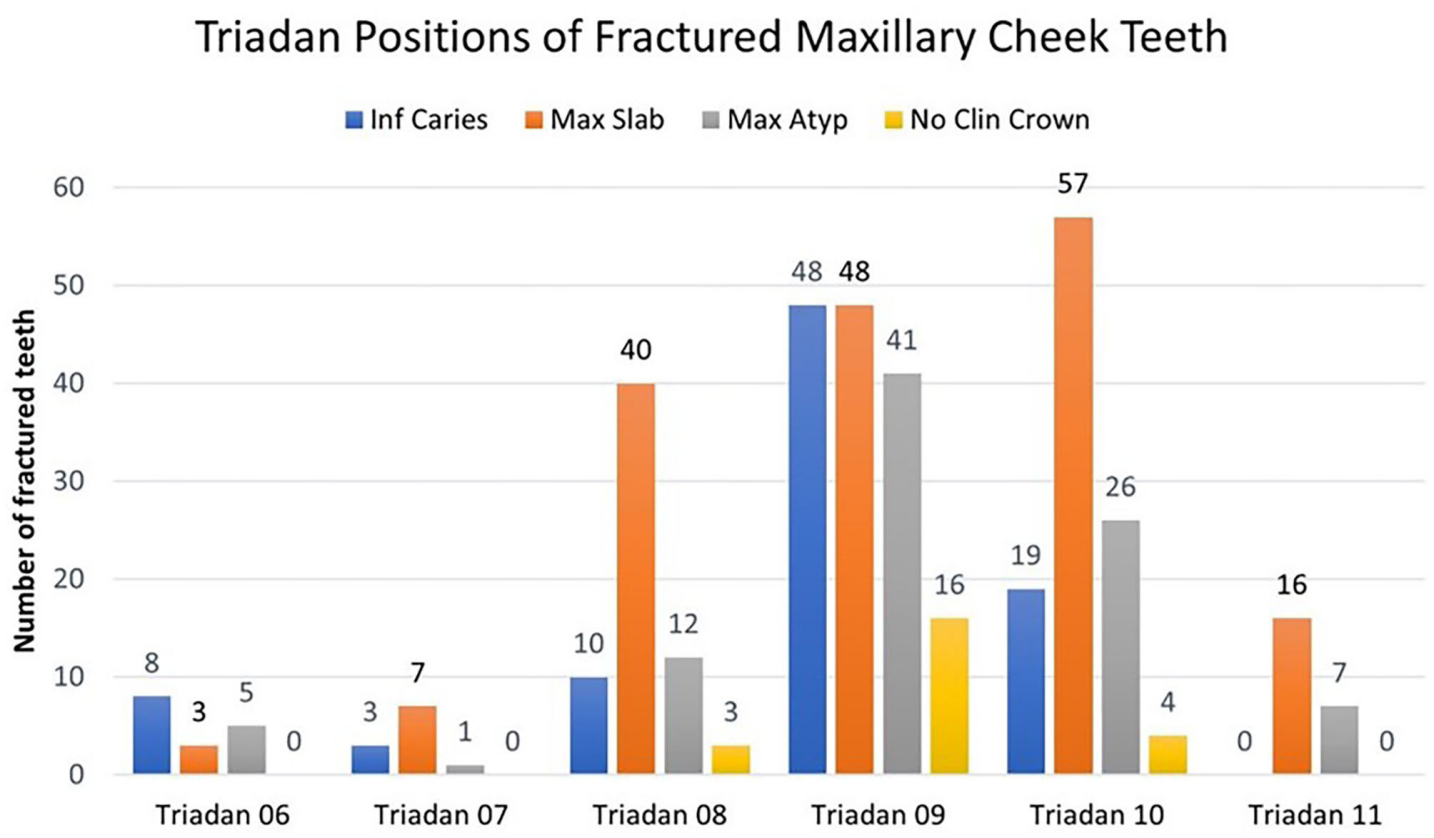
Triadan positions of 374 fractured maxillary cheek teeth with four different fracture patterns according to Triadan positions. Inf Caries, infundibular caries-related fracture; Max Slab, maxillary first and second pulp horn fractures; Max Atyp, atypical maxillary cheek teeth fracture patterns; No Clin Crown, fracture with the absence of clinical crown.

Most 374/486 (77%) fractures were in maxillary cheek teeth and the maxillary 09 position was preferentially affected, comprising 153/374 (41%) of all fractured maxillary teeth (Figure 6). These included 48/88 (55%) of infundibular caries-related fractures, with 19/88 (22%) of this fracture pattern in the Triadan 10 position, but none in the 11 position (Figure 6). The frequency of infundibular caries-related fractures differed significantly (*P* < 0.001) between tooth positions, with Triadan 09s significantly (*P* < 0.001) more (OR = 1.107, 95% CI = 1.06–1.19) likely to have this fracture than all other maxillary teeth combined.

Most (145/171; 85%) maxillary “slab” fractures occurred in the Triadan 08–10 positions, with the highest frequency (57/171; 33%) in the Triadan 10s. The original fracture pattern(s) in maxillary cheek teeth with complete loss of clinical crown is unknown. Although 16/23 (70%) of this fracture pattern were Triadan 09s, these are unlikely to have been originally caused by infundibular caries-related fractures, because these teeth did not have the full-depth, midline sagittal fractures characteristic of this disorder.

Overall, in all three types of “idiopathic” maxillary fractures (“slab”, “atypical,” or complete clinical crown loss) (*n* = 286), the Triadan 09s (37% of fractures), Triadan 10s (30%), and Triadan 08s (19%) were significantly (*P* < 0.001) more (OR = 1.12, 95% CI = 1.09–1.18) commonly affected than other three Triadan positions (Figure 6).

#### Mandibular Cheek Teeth

**Figure 7:**
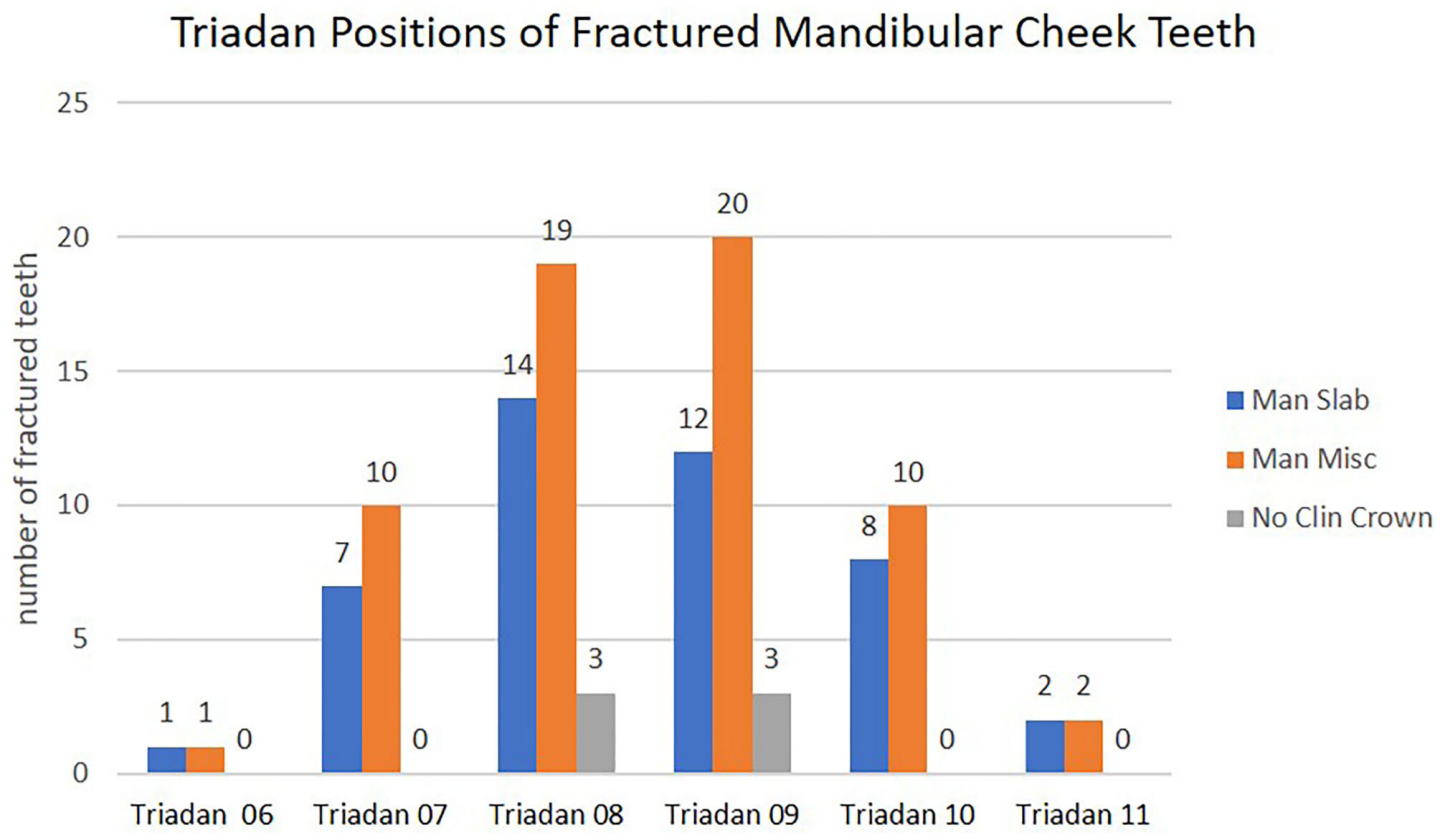
Triadan positions of 112 fractured mandibular cheek teeth with three different fracture patterns according to Triadan positions. Man Slab, mandibular first and second pulp horn fractures; Man Misc, atypical mandibular cheek teeth fracture patterns; No Clin Crown, fracture with the absence of clinical crown.

The frequency of idiopathic mandibular cheek teeth fractures varied significantly between tooth position (*P* < 0.001). The mandibular Triadan 08 and 09s were most commonly affected and comprised 71/112 (63%) of all mandibular dental fractures, with a reduced frequency in the 07 and 10s and the 06 and 11s rarely (5%) affected (Figure 7).

### Clinical Signs

**Figure 8:**
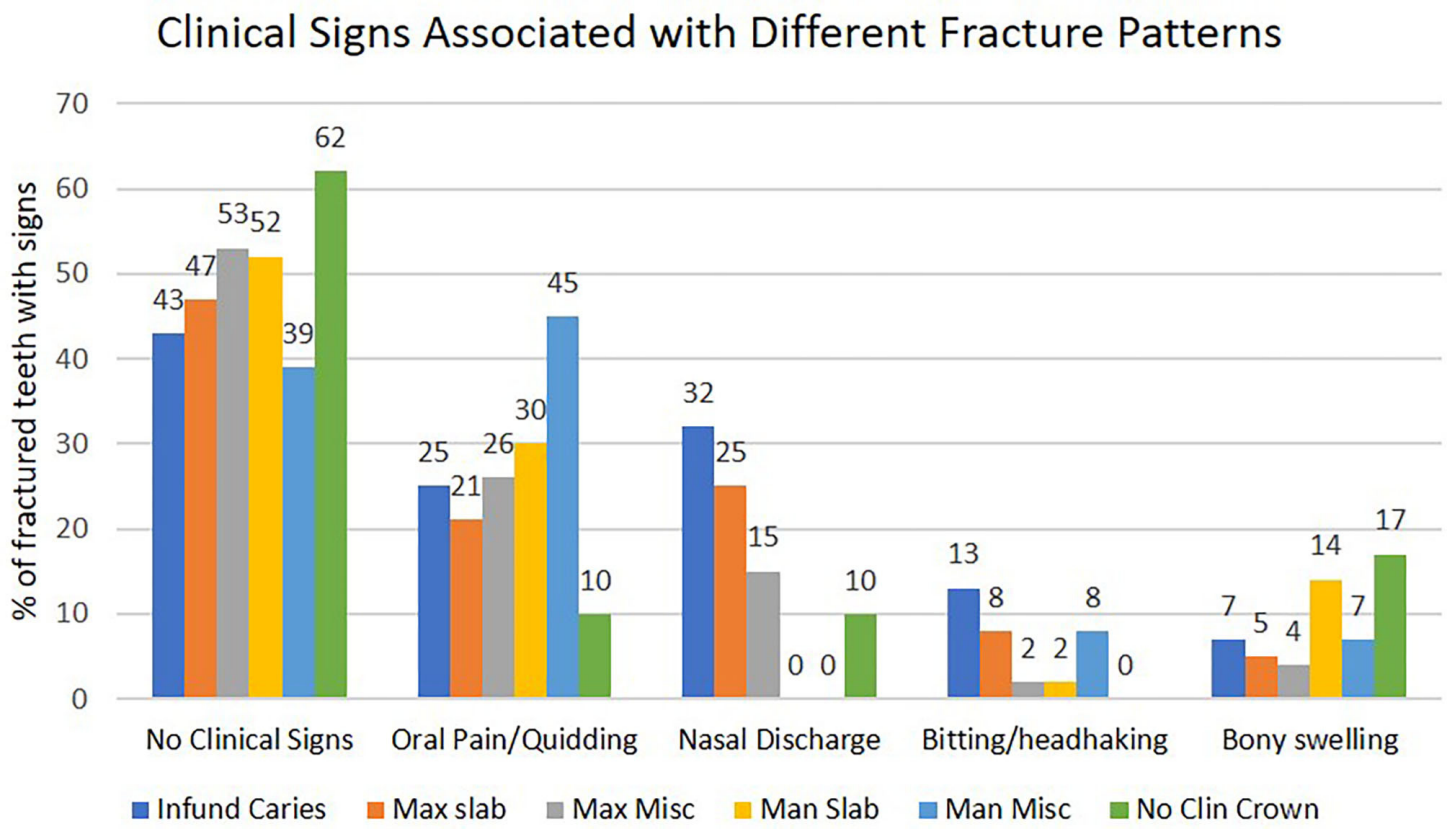
The presenting clinical signs in 300 horses with 486 cheek teeth fractures, subdivided into six different fracture patterns. Infund Caries, infundibular caries-related fracture; Max slab, maxillary first and second pulp horn fractures; Max Misc, atypical maxillary cheek teeth fracture patterns; No Clin Crown, fracture with the absence of clinical crown; Man Slab, mandibular first and second pulp horn fractures; Man Misc, atypical mandibular cheek teeth fracture patterns.

No clinical signs were noted in horses with 232/486 (47.7%) fractured teeth (Figure 8). Oral pain with dysmastication and quidding was the most common sign in the remainder and was recorded in horses with 126/486 (25.9%) of all fractured teeth. Bitting problems were present in horses with 26/486 (5.3%) fractured teeth. As noted, signs of quidding and bitting problems were combined as “oral pain” for statistical analysis. Atypical mandibular cheek teeth fractures were significantly (*P* < 0.001) more likely (OR = 3.31, 95% CI = 1.91–5.76) to be associated with oral pain and bitting problems (observed in 32/62 = 51.6%) than all other fracture types combined (105/42 = 24.8%). Fractures with complete clinical crown loss were associated with the lowest frequency (10%) of oral pain (Figure 8).

Unilateral nasal discharge was present with 88/374 (23.5%) of all fractured maxillary teeth and was significantly (*P* = 0.019) more common (OR = 1.9, 95% CI = 1.1–3.2) with infundibular caries-related fractures (28/88 = 31.8%) than with all other types of maxillary teeth fractures (combined 60/284 = 21.1%).

Maxillary (*n* = 20) or mandibular (*n* = 13) swellings were present with 33/486 (6.8%) of fractures (Figure 8). Clinical signs of apical infection were determined by the presence of ipsilateral unilateral nasal discharge (due to sinusitis in 86 horses and nasal infection in two cases—with 06/07 fractures) and/or the presence of maxillary or mandibular swellings caused by apical infection. Eight horses had both maxillary swellings and nasal discharge. Overall, clinical signs of apical infection were present in horses with 113/486 (23.3%) fractured teeth, varying from 4/62 (6.5%) teeth with atypical mandibular fractures to 32/88 (36.4%) teeth with caries-related infundibular fractures. Less common signs were weight loss in 13/486 (3%), headshaking with 6/486 (1.2%), and halitosis with 5/486 (1%) of fractured teeth.

### Treatment of Fractured Teeth

**Figure 9:**
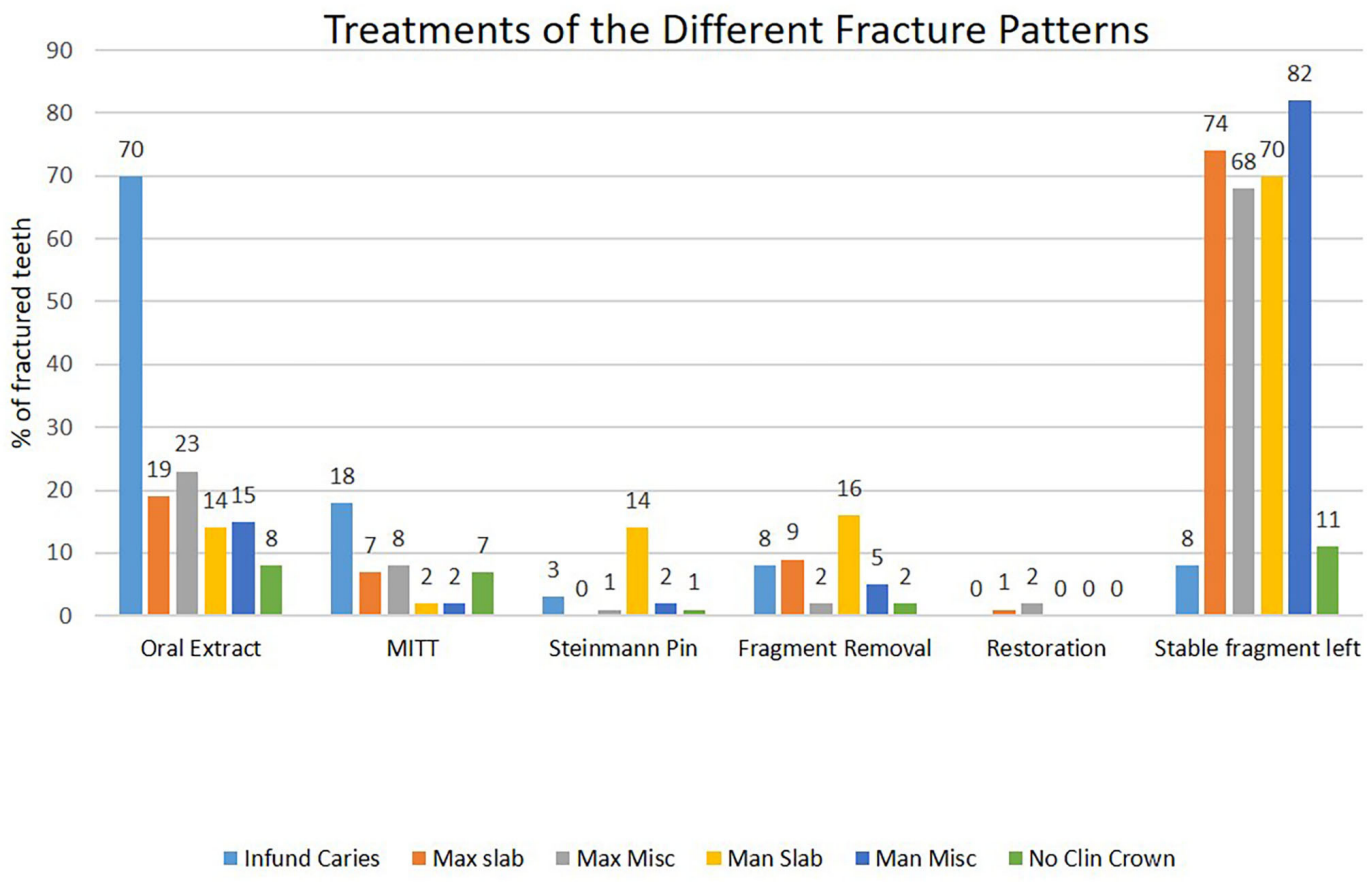
Treatments of 486 fractured cheek teeth with six different fracture patterns: including oral extraction (Oral Extract), minimally invasive transbuccal technique (MITT), Steinmann pin repulsion (Steinmann pin), fragment removal, dental restoration (Restoration), or stable fragment left *in situ*. Infund caries, infundibular caries-related fracture; Max slab, maxillary first and second pulp horn fractures; Atypic max, atypical maxillary cheek teeth fracture patterns; Man slab, mandibular first and second pulp horn fractures; Atypic man, atypical mandibular cheek teeth fracture patterns; No Clin Crown, fracture with the absence of clinical crown.

Treatment included extraction of 194/486 (40.0%) fractured teeth, or of all remaining fragments (Figure 9). Indications for complete extraction were teeth causing clinical apical infection, or where all remaining fragments were loose on manipulation with extraction forceps or grossly carious. Exodontia was by oral extraction in 138/194 (71%), including by the use of specialized dental picks when insufficient or no clinical crown remained. When oral extraction was not possible, Steinmann pin repulsion (*n* = 12) was used (especially in mandibular teeth with ventral drainage tracts) but later was largely superseded by the minimally invasive transbuccal technique (MITT) (*n* = 44).

Frequency of extractions differed significantly between fracture types (*P* < 0.001), with infundibular caries-related fractures extracted significantly more frequently (81/88; 92%) than all other types. In only 7/88 (8%) of infundibular caries-related fractures were a (single) stable fragment was left *in situ* in horses without clinical signs.

A conservative approach was taken for fractured teeth that were not causing clinical signs, including some teeth that later developed occlusal exposure of one or more of the remaining pulps. Conservative treatment included odontoplasty of sharp edges and of possible protruding aspects of the two adjacent (normal) teeth and of a few millimeters of the occlusal aspects of the fractured and of the opposite tooth. Over the period of this study, 3/127 conservatively treated maxillary “slab” fractures subsequently needed exodontia at this clinic due to development of clinical apical infection.

### Case Study of a Horse With Multiple Idiopathic Cheek Teeth Fractures

One horse, which was 7 years old at initial presentation, with multiple cheek teeth fractures, was examined 25 times over a 13-year period (including during the 8 years of this study). Over this time, the horse developed six maxillary and one mandibular idiopathic cheek teeth fractures, including five maxillary “slab” fractures, one initially presenting as a second pulp fracture later extending to the first pulp, one maxillary third and fourth pulp fracture, and one mandibular “slab” fracture. Over this period, clinical signs indicating possible dental disease were recognized in only five occasions. These included nasal discharge due to sinusitis, surprisingly with no dental involvement.

Sudden-onset oral pain/quidding for 1–2 days occurred on four occasions after maxillary buccal “slab” fractures developed and became displaced. These caused buccal trauma that immediately resolved following spontaneous loss or removal of these fragments and odontoplasty. The initial development of fractures caused recognized clinical signs with only one of the seven fractured teeth. The clinical signs on the other three occasions were caused by the development of new maxillary “slab” fractures some 5–8 years after recognition of the original fractures, following further clinical crown eruption. All seven teeth were treated by loose fragment removal and odontoplasty only.

Four of these seven teeth developed occlusal pulpar exposure and one later developed additional fracture patterns, but all four remained without signs of apical infection between 6 and 11 years at the last examination. Three other fractured teeth were of normal appearance, 9–11 years after their initial development of fractures.

## Discussion

Some earlier veterinary literature has descriptions of equine teeth affected with idiopathic and infundibular caries-related fractures, some of which were attributed to the presence of small stones in feedstuffs (9, 11). Dacre et al. (2) first described the presence of fractures through pulp horns that were present in 86% of idiopathic cheek teeth fractures. They showed chronic, pre-existing endodontic disease in 25% of these teeth that likely predisposed to fractures. Fractures through infundibula were found in the remaining 14% of teeth (2). As noted, this midline sagittal fracture pattern now has an identified etiology and can no longer be termed “idiopathic” and has been termed infundibular caries-related fracture (8).

### Number of Fractured Teeth per Horse

This study found a mean of 1.6 fractured teeth per affected horse, while previous referral hospital (3) and general practice (4) surveys in 2007 found 1.1 and 1.2 fractured teeth per horse, respectively. The increased prevalence in the current study is likely due to improved recognition of this disorder as well as to the increased use of oral endoscopy.

### Maxillary Teeth Fracture Patterns

Maxillary cheek teeth were most commonly (77%) fractured in this study (Figure 6), which is similar to previous findings of 82% (1), 60% (2), 82% (3), and 58% (4) maxillary involvement. Caries-related infundibular fractures comprised 23.5% of maxillary dental fractures in this study, similar to the findings of previous referral and general practice studies (both 23%) (3, 4).

The most common maxillary (and overall) dental fracture pattern found in this study was a “slab” (first and second pulp horns) fracture that comprised 46% of all maxillary dental fractures. This was also the most common maxillary dental fracture pattern reported in a pathological study (comprised 43% of maxillary fractures) (2), referral hospital study (45%) (3), and general practice study (48%) (4).

Twenty-eight other patterns of maxillary cheek teeth fractures were found in 92 teeth, i.e., “atypical” fracture patterns, including the six shown in Figure 3. These fracture planes traversed single pulp horns in 63% of these “atypical” fractures. The first pulp horns were involved (alone or with other pulps or infundibula) in 46% of “atypical” fractures and the second pulp horn in 50%, as compared to a mean of 31% involvement of the three palatal (third to fifth) maxillary pulp horns. Of interest is that 8.7% (8/92) of these atypical maxillary fractures had a fracture plane running through a single infundibulum in addition to one or more pulp horns. There was evidence of progression of individual first or second pulp horn (“atypical”) fractures to become conjoined first and second pulp horn (“slab”) fractures in some teeth.

### Mandibular Teeth Fracture Patterns

The most common mandibular cheek teeth fracture pattern (comprising 42% of mandibular dental fractures) was also a first and second pulp horn (“slab”) fracture (Figure 4), which is a slightly lower proportion than found in a previous referral (57%) (3) or a general practice (53%) (4) study. Most equine fissure (hairline) cheek teeth fractures are transversely oriented (in a bucco-palatal/lingual direction) and seldom cause clinical disease (12–14). However, two studies have shown that sagittal fissure fractures involving both first and second pulp horns can later cause complete (complicated) first and second pulp horn fractures (15, 16). Pollaris et al. (17) have shown progression of fissure fractures to complete but non-complicated fractures, but Wellman and Dixon (18) have shown pulpar exposure and infection with deep fissure fractures. Sixteen other “atypical” mandibular cheek tooth fracture patterns involving 62 teeth were also described in this study (Figure 5), with 61% through a single pulp horn.

### Complete Clinical Crown Loss

Twenty-nine teeth with complete loss of clinical crown, some with caries of the remaining reserve crown, were also identified in this study. Many involved the maxillary 09s, but none as noted had deep sagittal fractures and, thus, were unlikely to have been originally caused by caries-related infundibular fractures. In retrospect, this fracture pattern could possibly have been included in the “atypical” fracture group.

### Age of Affected Horses

Horses with infundibular caries-related fractures (median 15 years of age) were significantly older than horses with maxillary slab fractures (median 11 years). A similar age differential (median ages of 12 vs. 10 years) was found in a previous study but was not statistically significant, possibly due to low numbers of cases (3). The age of horses with infundibular caries-related fractures is likely related to when infundibular cemental defects in these teeth (many of which are located deep in the infundibulum) become occlusally exposed and develop infundibular caries (5–7, 19).

However, the reason for the relatively mature age of horses with idiopathic fracture patterns (mean 11.9–13.5 years) dependent on the fracture pattern is unclear. In idiopathic fractured teeth with pre-existing endodontic disease (2), it may be due to the slow progression of this disease that prevents deposition of new secondary circumpulpar dentine and also gradually weakens the integrity of dentine. In most (other) teeth without pre-existing endodontic disease, it would seem more logical that such fractures should develop in much younger teeth that have less secondary dentine and thus larger pulp horns than in mature teeth, such as what was found in this study (median age 11–13 years for “idiopathic” fractures).

### Triadan Position of Fractured Teeth

The maxillary 09s were the most commonly fractured tooth position (comprised 41% of all maxillary dental fractures). This can be partly explained by the recognized predisposition of Triadan 09s to develop infundibular caries-related fractures (5–7, 15, 19). In this study, 55% of this fracture pattern was in the 09 position. However, the 09s as well as the 08s and the 10s were also commonly affected with all other types of maxillary dental fractures (Figure 6) as previously found (2–4, 8). The other, more peripheral Triadan positions including the Triadan 11s were uncommonly fractured, despite the highest occlusal pressures being in the teeth closest to the temporo-mandibular joint, i.e., the 11s (20).

Most mandibular cheek teeth fractures involved the two most centrally located teeth (08s and 09s) as previously reported (3, 4). There was a decreasing prevalence of fractures toward the peripherally situated teeth, and fractures were rarely found in the 06 and 11s (2 and 4%, respectively, of all mandibular teeth fractures) (Figure 7). Both studies also seldom found mandibular Triadan 10 fractures (3, 4).

### Clinical Signs

No clinical signs were noted in horses with 48% of fractured teeth. This high proportion without signs may reflect current better recognition of these disorders and improved diagnostic techniques. In contrast, an earlier study found that every fractured tooth caused clinical signs, including apical infections in 50% of cases (1). Oral pain with dysmastication and quidding was the most common recognized sign in this study (associated with 26% of fractures) and was most common in horses with “atypical” mandibular fractures (45% incidence). Oral pain/quidding was least common (10%) with fractures with total loss of the clinical crown—which therefore could not lacerate the soft tissues. This fracture pattern also had minimal masticatory-induced movement of the remaining crown that could overstretch the periodontal ligaments. Bitting and headshaking problems, also likely due to oral discomfort, were present in 7% of cases, which is inexplicably much lower than previous referral (28% incidence) (3) and practice (29%) (4) studies.

Signs of apical infection as determined by nasal discharge or swelling of the supporting bones was present with 23% of dental fractures. It was more common with infundibular caries-related fractures (36% incidence) and maxillary slab fractures (27%) and lowest with mandibular “slab” (14%) and “atypical” mandibular (7%) dental fractures. These findings are similar to a referral hospital study that found 21% of fractured teeth to have clinical apical infection (3) and, as expected, much greater than a general practice survey that found just 3% of fractures to have clinical apical infection (4). However, the findings of these three studies differ greatly from a recent CT study by Rowley et al. (10) that showed 77% (62/81) of all fractured cheek teeth, including 80% of maxillary cheek teeth and 43% of mandibular cheek teeth, have CT evidence of apical infection. In that study, all infundibular caries-related fractures and 73% of maxillary “slab” fractures had apical changes. These findings would suggest that a high proportion of asymptomatic fractured teeth in the current study also had apical infection that were confined to the alveolus or did not necessarily cause generalized pulpar death.

### Treatments

All teeth causing clinical apical infection (i.e., nasal discharge, sinus tracts, or gross bony swellings) or where all dental fragments were loose or very carious were extracted. Exodontia was *per os* with forceps and picks where possible. Teeth with infundibular caries-related fractures had the highest level of clinical apical infection, and thus, 92% (81/88) of such teeth were extracted. If MITT had been available earlier in this study, four of the seven cases where dental fragments were left would likely have had all fragments extracted. Lower proportions of teeth with other fracture patterns met the above criteria for extraction and so fewer were extracted.

There is no consensus regarding the treatment of fractured teeth that are not causing clinical signs. A conservative approach to treatment was adopted in this, as in a previous referral hospital study (3). Thus, larger stable dental fragments (e.g., 74% of maxillary cheek teeth with loss of “slab” fractures) were left *in situ*. It was hoped that any exposed viable pulps would become sealed off with tertiary dentine or, alternatively, that any possible pulpar and apical infection of the remaining tooth would be walled off within a sclerotic alveolus and prevent clinical signs of apical infection from developing.

At least 3/171 (2%) conservatively treated maxillary teeth with “slab” fractures later developed clinical apical infection and required exodontia at this clinic. It is possible that other conservatively treated horses later developed such signs and were treated elsewhere.

A smaller referral study found a higher proportion of 6/22 (27%) of conservatively treated fractured teeth to later require exodontia (3).

In the current study, some conservatively treated fractured teeth later developed occlusal exposure of the remaining pulp horns indicating death of these pulp horns (and thus of the tooth) without clinical signs of apical infection. There is increased rationale for the extraction of such teeth, or at least monitoring them by imaging. However, as noted, a CT study found that 77% of fractured teeth have imaging evidence of apical infection, although not necessarily confirmative of pulpar death (10). The rationale for extraction of such a high proportion of fractured teeth (e.g., one horse in this study with 10 fractured teeth) would not have been justified. The endodontic treatment of equine cheek teeth has been recently reported (21) and will likely be increasingly used in the future. However, there is difficulty in gaining suitable access to the most common idiopathic fracture pattern (i.e., maxillary “slab” fractures) that would likely benefit from endodontic treatment, but some clinicians have overcome this technical problem (Lundström, 2018, personal communication).

Extractions of multiple fractured teeth, such as maxillary slab fractures (often Triadan 10s), in young mature horses can be technically difficult, with further extraction-related fractures occurring. In fact, postextraction sequelae may result in protracted clinical disease in horses without previous clinical signs. Conservative treatments, such as those performed on one horse with seven fractured teeth and examined 25 times over 13 years, were found to be satisfactory for that horse's welfare and for that client's expectations. Extraction of all seven fractured teeth would have been far more traumatic for this horse and also would have reduced its ability to masticate effectively.

There may also be financial incentives to extract teeth that have endodontic or apical changes. The annual renewal of a horse's veterinary care insurance may place pressure for dental extraction prior to the renewal date, in case dental fractures are excluded in future policies. Perhaps, equine health insurers need to take a longer term (and possibly less expensive) view on such cases that will also be in the horse's welfare interests.

## Conclusions

Non-traumatic cheek teeth fractures are increasingly recognized in horses, with almost half of the fractures in this study in horses with no clinical signs. Fractured teeth with apical infection (which includes most teeth with infundibular caries-related fractures) and those with multiple fractures or advanced caries require extraction. Most other teeth do not necessarily need immediate extraction, and most can remain *in situ* for many years without causing clinical signs, despite the presence of subclinical endodontic and apical changes in some. Further studies of these disorders with repeat imaging and obtaining long-term follow-up information of all cases would be of value.

## Data Availability Statement

The raw data supporting the conclusions of this article will be made available by the authors, without undue reservation.

## Author Contributions

PD contributed to study design and execution, data analysis and interpretation, and manuscript preparation. RK contributed to study execution and manuscript preparation. RR contributed to data analysis and interpretation, and manuscript preparation. All the authors approved the final manuscript.

## Conflict of Interest

The authors declare that the research was conducted in the absence of any commercial or financial relationships that could be construed as a potential conflict of interest.
